# Increased STX3 transcript and protein levels were associated with poor prognosis in two independent cohorts of esophageal squamous cell carcinoma patients

**DOI:** 10.1002/cam4.6770

**Published:** 2023-11-28

**Authors:** Takahiro Shinozuka, Mitsuro Kanda, Yusuke Sato, Dai Shimizu, Chie Tanaka, Shinichi Umeda, Yoshikuni Inokawa, Norifumi Hattori, Masamichi Hayashi, Goro Nakayama, Yasuhiro Kodera

**Affiliations:** ^1^ Department of Gastroenterological Surgery Nagoya University Graduate School of Medicine Nagoya Japan; ^2^ Department of Thoracic Surgery Akita University Graduate School of Medicine Akita Japan

**Keywords:** biomarker, esophageal squamous cell carcinoma, Syntaxin 3

## Abstract

**Background:**

Some conventional prognostic biomarkers for esophageal squamous cell carcinoma (ESCC) have the disadvantage that they have only been investigated at the level of either mRNA or protein levels or only in individual cohorts. Associations between Syntaxin 3 (STX3) expression and malignancy have been reported in several tumor types but not in ESCC. Here, we investigated the levels of both STX3 mRNA and protein, and its prognostic potential in two independent cohorts of patients with ESCC.

**Methods:**

*STX3* mRNA levels were examined in surgical specimens by quantitative PCR in a cohort that included 176 ESCC patients. STX3 protein levels were investigated in surgically resected ESCC tissues by immunohistochemistry using tissue microarrays in a different cohort of 177 ESCC patients. Correlations were analyzed between the expression of STX3 mRNA and protein with clinicopathological factors and long‐term prognosis.

**Results:**

Quantitative PCR indicated a significant association between high level of STX3 mRNA expression and lymph node involvement, pathological stage, and poor overall survival. The multivariate analysis demonstrated that high *STX3* mRNA expression was independently associated with poor overall survival outcomes. Immunohistochemistry revealed that STX3 protein expression in ESCC tissues and high STX3 protein expression were also significantly correlated with unfavorable overall survival.

**Conclusions:**

Overexpression of STX3 mRNA and protein may serve as potential prognostic biomarkers for ESCC patients.

## Graphical abstract

High STX3 mRNA and protein levels in surgical specimens were significantly correlated with poor prognosis in two independent cohorts of esophageal squamous cell carcinoma patients. STX3 overexpression in esophageal squamous cell carcinoma tissues may be a prognostic biomarker.

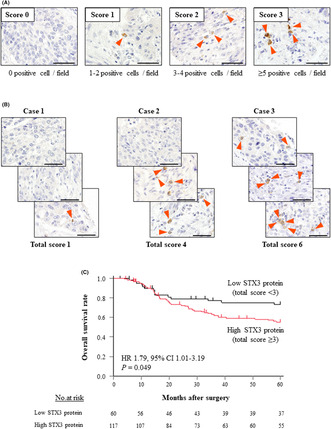

## 1 INTRODUCTION

Esophageal cancer ranks as the sixth leading cause of cancer‐related mortality globally.^1^ In Asian countries, the predominant histopathological type is esophageal squamous cell carcinoma (ESCC).^2^ In spite of progress in perioperative multidisciplinary treatment for ESCC, the prognosis of patients after radical esophagectomy remains poor.^3^ Thus, there is a need to determine precise prognostic biomarkers for ESCC after radical treatment, which could help in optimizing postoperative treatment and follow‐up plans for individual patients.

However, previously reported prognostic biomarkers for ESCC have had drawbacks in their study design, such as single cohort analysis or a focus on either gene or protein expression, which could limit their predictive power and validity.^4^, ^5^, ^6^ To overcome these limitations, it is important to combine gene expression and protein expression data and to validate findings in multiple independent cohorts.

Members of the syntaxin gene family have transmembrane domains that are required for membrane fusion, are localized to the plasma membrane, endoplasmic reticulum, and Golgi apparatus, and are involved in intracellular vesicular reticulum trafficking.^7^, ^8^, ^9^, ^10^, ^11^ Syntaxin 3 (STX3) is involved in exocytosis^12^, ^13^ and has been reported to promote cancer cell proliferation in breast cancer^14^ and to predict survival outcome in lung cancer,^15^ but no study has reported its role in ESCC.

This study assessed the significance of STX3 mRNA and protein expression for prognosis in patients with ESCC. For this purpose, we first assessed the correlation between *STX3* mRNA expression in ESCC tissue samples and long‐term prognosis in one cohort. Next, we performed immunohistochemistry (IHC) of tumor tissue microarrays (TMAs) obtained in another set of ESCC patients to examine the prognostic significance of STX3 protein expression.

## 2 METHODS

### 2.1 Ethics

This study adheres to the ethical standards outlined in the Declaration of Helsinki by the World Medical Association regarding human subject research and received approval from the Ethics Committee of Nagoya University Hospital, Japan (approval number 2014‐0043). Every participant gave their written consent for the utilization of their clinical data and samples, as mandated by the Institutional Review Board.

### 2.2 STX3 mRNA dataset

For the STX3 mRNA dataset, we obtained 176 primary ESCC tissue samples from surgically resected specimens of patients undergoing curative esophagectomy at Nagoya University Hospital from 2001 to 2016. After resecting tissue samples, we promptly submerged them liquid nitrogen and preserved them at −80°C. Patients diagnosed with clinical Stage II‐III according to the 8th edition of the Union for International Cancer Control (UICC) 8th edition,^16^ underwent neoadjuvant chemotherapy (NAC) unless contraindicated.^17^ The postoperative follow‐up strategy entailed regular physical assessments, blood tests including serum tumor markers, and contrast‐enhanced CT scans quarterly, along with yearly upper gastrointestinal endoscopy, all for a duration of 10 years.^18^

### 2.3 Quantitative real‐time PCR (qPCR)

To analyze *STX3* mRNA expression levels in clinical specimens from ESCC patients and ESCC cell lines, qPCR was performed as previously described.^19^, ^20^ Glyceraldehyde‐3‐phosphate dehydrogenase (*GAPDH*) mRNA served as the internal standard in this study,^21^ and expression levels in each sample were measured in triplicate and calculated as STX3 mRNA divided by GAPDH mRNA. The primers employed for qPCR are detailed in Table S1.

### 2.4 STX3 protein dataset

For the STX3 protein dataset, we collected 177 primary tissue samples of ESCC patients who received radical esophagectomy at Akita University Hospital, Japan from 2000 to 2011.^22^ These patients did not receive any treatment before curative surgery.

### 2.5 TMA analysis

To investigate STX3 protein expression in ESCC tissues by IHC, tissue samples were embedded in paraffin, and TMAs were established. TMAs incorporated three punched specimens from each patient to reduce variability in tumor tissue.^23^ TMA blocks were incubated with a 1:2000 dilution of mouse anti‐STX3 polyclonal antibody (66760‐1‐Ig; Proteintech, Chicago, IL, USA) using antibody diluent, and incubated for 1 h at room temperature. To evaluate tissue staining, two researchers, who were not informed of the patients' clinical data, determined scores as follows: STX3 protein expression in the field with the most stained cells (1000× magnification) was scored as 3 (≥5 positive cells/field), 2 (3–4 positive cells/field), 1 (1–2 positive cells/field), and 0 (0 positive cells/field; no staining).

### 2.6 Cell lines

A total of 21 ESCC cell lines, specifically KYSE30, KYSE70, KYSE140, KYSE150, KYSE180, KYSE270, KYSE410, KYSE450, KYSE510, KYSE590, KYSE890, KYSE1170, KYSE1260, KYSE1440, NUEC2, TE1, TE2, TE3, WSSC, TT, and TTn, along with Het‐1A, a non‐cancerous epithelial cell line, were purchased from the Japanese Collection of Research Bioresources Cell Bank (Osaka, Japan), the American Type Culture Collection (Manassas, VA, USA), or were established at Nagoya University.^5^ The cells were cultured in a humidified incubator at 37°C with an atmosphere composition of 5% CO_2_ using RPMI‐1640 (Sigma‐Aldrich, St. Louis, MO, USA) supplemented with 10% fetal bovine serum and antibiotics.^24^

### 2.7 Small interfering RNA (siRNA)‐mediated knockdown of STX3

KYSE70 cells, at a density of 2 × 10^5^ per well, were plated into 6‐well plates, transfected with control (siControl) or STX3‐specific (siSTX3) siRNAs, cultured in serum‐free RPMI‐1640 medium for 48 h prior to their utilization in western blot analysis and Simple Western assays. The efficiency of KLRG2 knockdown was determined by qPCR as described above. The sequences of siRNA are provided in Table S1.

### 2.8 Western blot analysis and Simple Western assays

For evaluation of STX3 protein levels, cell lysis, SDS‐PAGE, and protein transfer to membranes were performed according to standard procedures. Following the blocking step, the blots were incubated with a mouse anti‐STX3 monoclonal antibody (66760‐1‐Ig; Proteintech, Rosemont, IL, USA) at a 1:1000 dilution, and subsequently with horseradish peroxidase‐conjugated anti‐mouse IgG secondary antibody (#7076; Cell Signaling Technology, Tokyo, Japan). The blots were treated with Can Get Signal Solution (NKB‐101; TOYOBO, Osaka, Japan) and visualized using ChemiDoc MP (Bio‐Rad, Hercules, CA, USA).

To analyze proteins expressed at levels correlating with STX3 expression, proteins associated with major cancer‐related signaling pathways were analyzed by the Simple Western system (ProteinSimple, San Jose, CA, USA). In this assay, 3 μg of total protein extracted from KYSE70 cells was probed using the following primary monoclonal antibodies from Cell Signaling Technology, each at a 1:50 dilution: PI3 Kinase Antibody Sampler Kit (#9655), Phospho‐Akt Pathway Antibody Sampler Kit (#9916), mTOR Substrate Antibody Sampler Kit (#9862), Phospho‐Erk1/2 Pathway Antibody Sampler Kit (#9911), Wnt Signaling Antibody Sampler Kit (#2915) and β‐catenin Antibody Sampler Kit (#2951). The levels of protein expression were normalized to β‐actin levels and the data were processed with Compass for Simple Western software (ProteinSimple).

### 2.9 Statical analysis

The Mann–Whitney test was used for continuous variables, while the chi‐squared test was applied to categorical variables to evaluate differences between two groups. Survival curves were generated using the Kaplan–Meier method. Overall survival (OS) was defined as the time from the day of surgery to the day of death due to any cause, and disease‐free survival (DFS) was defined as the time from the day of surgery to the day of any disease event, which includes recurrence of esophageal squamous cell carcinoma (ESCC).^25^ The Cox proportional hazards model was employed to calculate hazard ratios (HR) and 95% confidence intervals (CIs) and to conduct multivariate regression analysis for potential prognostic markers. The multivariate analysis incorporated variables that exhibited *p* < 0.05 in the univariate analysis. All statistical analyzes were performed using JMP 16 software, (SAS Institute, Cary, NC, USA). A *P* value below 0.05 was established as the criterion for statistical significance.

## 3 RESULTS

### 3.1 Correlations between STX3 mRNA levels and clinicopathological factors and prognosis

In the *STX3* mRNA dataset (*n* = 176), the median age of the patients was 65.5 years with an age range of 44–83 years. The male to female ratio was 137:39. In total 92 patients (52.2%) received NAC, and patients diagnosed with UICC pathologic Stage I, II, III, and IV were 29, 46, 91, and 10, respectively. Patients were categorized into two groups based on the third quartile cutoff of *STX3* mRNA expression levels in tumor tissue, for the purpose of evaluating the prognostic value of *STX3* mRNA expression. The low *STX3* mRNA group had 132 patients and the high *STX3* mRNA group had 44 patients. High *STX3* mRNA expression in tumor tissues showed a significant correlation with pathologic lymph node involvement and advanced pathologic stage when analyzing the association between *STX3* mRNA expression and clinicopathologic factors (Table 1).

**TABLE 1:** Association between the expression of *STX3* mRNA and clinicopathological parameters of 176 patients with esophageal squamous cell carcinoma.

Parameters	Low *STX3* mRNA (*n* = 132)	High *STX3 mRNA* (*n* = 44)	*p*
Age			
<65 years	61	18	0.540
≥65 years	71	26	
Sex			
Male	103	34	0.916
Female	29	10	
Preoperative symptoms			
Absent	34	7	0.181
Present	98	37	
Brinkman index			
<500	51	17	0.590
≥500	77	21	
CEA (ng/mL)			
≤5	115	39	0.792
>5	17	5	
SCC (ng/mL)			
≤1.5	86	22	0.079
>1.5	44	21	
Tumor size			
<5.0 cm	76	24	0.687
≥5.0 cm	55	20	
Tumor location			
Ut/Mt	80	28	0.721
Lt/Ae	52	16	
Neoadjuvant chemotherapy			
Absent	68	16	0.081
Present	64	28	
Pathological T factor			
T1/T2	51	17	1.000
T3/T4	81	27	
Lymph node metastasis			
Absent	52	10	0.045
Present	80	34	
Pathological stage			
I/II	63	12	0.018
III/IV	69	32	
Tumor differentiation			
Differentiated	113	38	0.901
Undifferentiated	19	6	
Lymphatic involvement			
Absent	36	10	0.552
Present	96	34	
Vessel invasion			
Absent	78	29	0.422
Present	54	15	
Intraepithelial spread			
Absent	97	34	0.618
Present	35	10	
Intramural metastasis			
Absent	122	41	0.868
Present	10	3	

Abbreviations: CEA, carcinoembryonic antigen; SCC, squamous cell carcinoma‐related antigen; *STX3*, Syntaxin 3.

**FIGURE 1:**
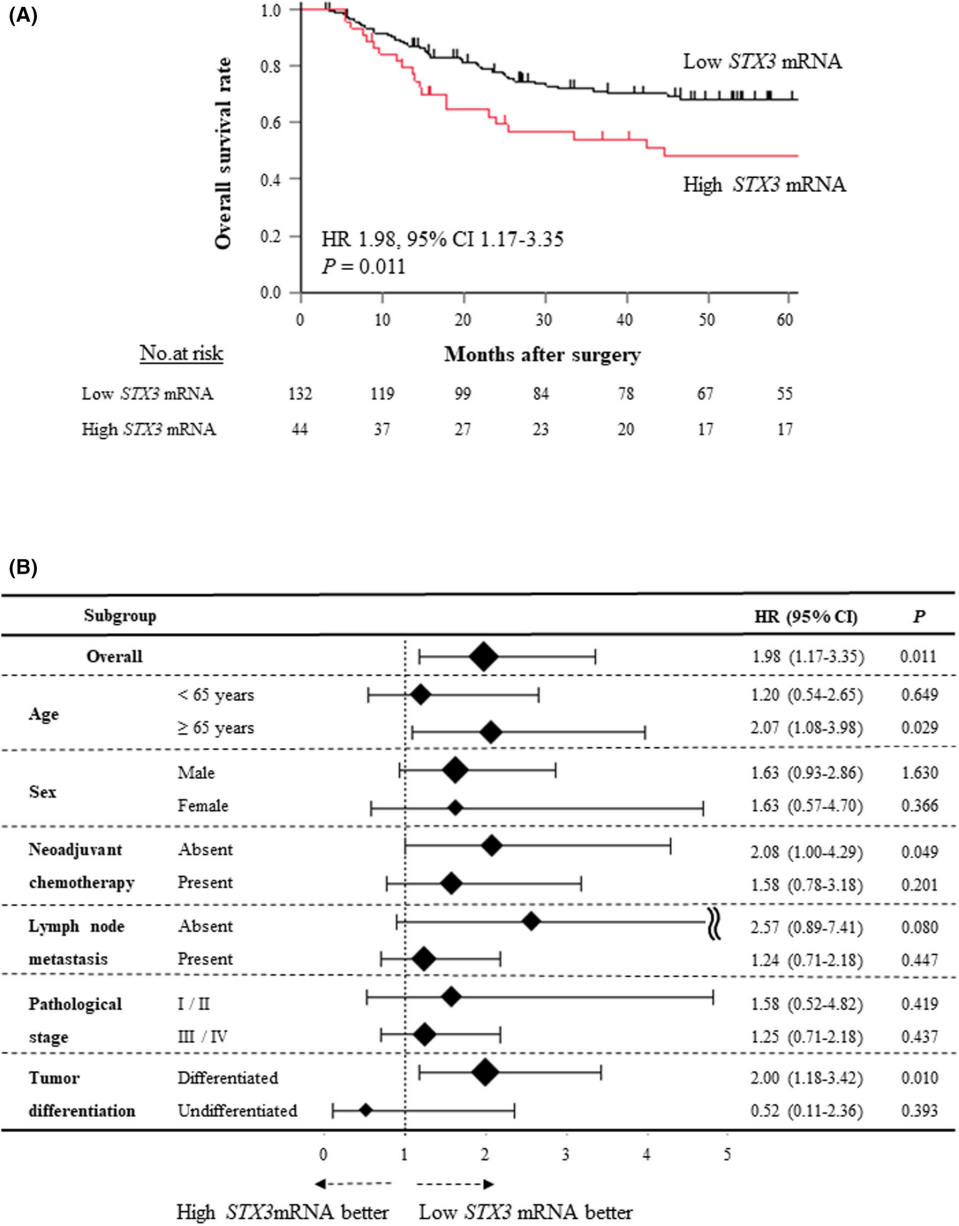
(A) Kaplan–Meier analysis of overall survival for the 176 esophageal squamous cell carcinoma patients after radical esophagectomy in the *STX3* mRNA dataset divided into two groups in accordance with *STX3* mRNA expression. (B) Subgroup analysis demonstrating the predictive value of *STX3* mRNA expression for overall survival.

OS was significantly shorter in the group with high *STX3* expression compared to the group with low *STX3* expression (HR: 1.98, 95% CI: 1.17–3.35, *p* = 0.011; Figure 1A). DFS was not significantly different between the two groups (HR: 1.56, 95% CI: 0.95–2.45, *p* = 0.079; Figure S1A). When analyzing initial recurrence patterns, there were no significant differences between the two groups regarding overall, nodal, local, or hematogenous recurrence (Figure S1B). Subsequently, we examined a publicly accessible database, the Kaplan–Meier plotter (https://kmplot.com/analysis/index.php?p=background), which utilizes the RNA sequencing data from 81 ESCC patients. The patients were classified into two groups based on *STX3* mRNA expression levels: low expression (*n* = 31) and high expression (*n* = 50), using a cutoff value of 1.0 (log). Patients with high *STX3* mRNA expression showed a tendency towards reduced OS compared to those with low *STX3* mRNA expression, however, the difference in OS between the two groups was not statistically significant (HR: 1.54, 95% CI: 0.61–3.89, *p* = 0.361; Figure S2).

We next performed a subgroup analysis according to age, sex, the administration of NAC, lymph node involvement, pathological stage, and tumor differentiation status to assess the impact of STX*3* mRNA expression levels on the prognosis of OS. We found significant differences in patients ≥65 years, those who received NAC, and the differentiated tumor groups (Figure 1B). Multivariate analysis demonstrated that high *STX3* mRNA expression was an independent prognostic factor for OS (HR: 1.80, 95% CI: 1.05–3.08, *p* = 0.034), as were lymphatic involvement, advanced pathological stage, and intramural metastasis (Table 2).

**TABLE 2:** Prognostic factors for overall survival in esophageal squamous cell carcinoma patients in *STX3* mRNA dataset (*n* = 176).

Variables	*n*	Univariate			Multivariable			
		Hazard ratio	95% CI	*p*	Hazard ratio	95% CI		*p*
Age (≥65 years)	97	1.07	0.65–1.74	0.801				
Sex (male)	137	0.98	0.55–1.74	0.935				
Preoperative symptoms	135	1.75	0.93–3.27	0.081				
Brinkman index (≥500)	98	0.95	0.56–1.61	0.842				
CEA (>5 ng/mL)	22	1.44	0.74–2.83	0.286				
SCC (>1.5 ng/mL)	65	1.56	0.94–2.57	0.083				
Tumor size (≥5.0 cm)	75	1.31	0.80–2.13	0.280				
Tumor location (Lt/Ae)	68	1.00	0.60–1.65	0.994				
Neoadjuvant chemotherapy	92	0.83	0.52–1.32	0.439				
Pathological T factor (T3/T4)	108	1.40	0.84–2.33	0.194				
Lymph node metastasis	114	3.55	1.86–6.80	<0.001	1.96	0.95–4.05		0.070
Pathological stage (III/IV)	101	3.63	2.13–6.20	<0.001	3.88	1.60–9.41		0.002
Tumor differentiation (poor)	25	1.24	0.63–2.44	0.526				
Lymphatic involvement	130	5.74	2.30–14.31	<0.001	5.02	1.74–14.45		0.003
Vessel invasion	69	1.52	0.93–2.48	0.091				
Intraepithelial spread	45	1.52	0.90–2.55	0.116				
Intramural metastasis	13	2.36	1.17–4.80	0.017	2.20	1.07–4.54		0.033
High *STX3* mRNA expression	44	1.98	1.17–3.35	0.011	1.80	1.05–3.08		0.034

Abbreviations: CI, confidence interval; CEA, carcinoembryonic antigen; SCC, squamous cell carcinoma‐related antigen; STX3, syntaxin 3.

### 3.2 Correlations between STX3 protein levels and clinicopathological variables and prognosis

**FIGURE 2:**
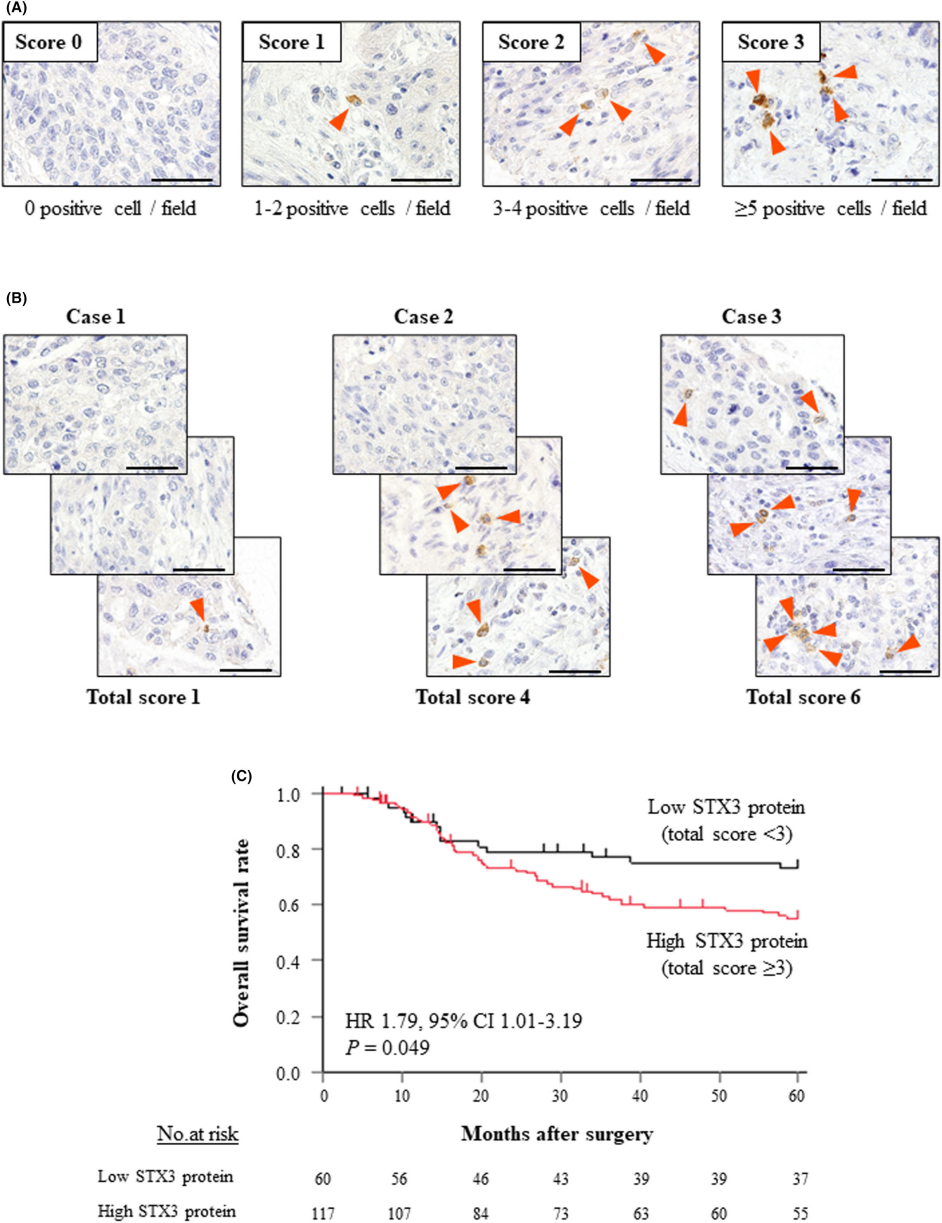
(A) Representative images of immunohistochemical staining for STX3 protein expression in TMAs containing samples from the STX3 protein dataset. Examples of scores 0 (0 positive cells/field; negative), 1 (1–2 positive cells/field), 2 (3–4 positive cells/field), and 3 (≥5 positive cells/field). Scale bars, 50 μm. (B) Representative images of cases included in the tumor microarrays. Total scores from the three samples were combined to obtain the final score for each case. Scale bars, 50 μm. (C) With the total from the three scores, the high STX3 protein group was defined as scores of ≥3 or more, and the low STX3 protein group was defined as scores of <3. Kaplan–Meier analysis of overall survival among 177 esophageal squamous cell carcinoma patients after radical esophagectomy in the STX3 protein dataset. Patients were divided into two groups in accordance with the levels of STX3 protein expression.

In the STX3 protein dataset (*n* = 177), the median age of the patients was 66 years, with an age range of 38–82 years. The male to female ratio was 153:24. There were 10, 44, 79, and 44 patients diagnosed with UICC pathologic Stage I, II, III, and IV, respectively. IHC analysis of STX3 protein expression in ESCC tissues using TMAs was assessed according to the scoring system detailed in the Methods section. Representative photomicrographs with scores of 0, 1, 2, and 3 are shown in Figure 2A. The final score for each patient was the sum of three samples (Figure 2B). To determine the prognostic value of STX3 protein expression in tumor tissues, patients were divided into two groups based on a total staining score cutoff of 3. This division created an approximate 2:1 ratio of patients with high versus low STX3 protein expression. There were 60 patients in the low STX3 group (total score <3) and 117 in the high STX3 group (total score ≥3), respectively. Analysis of associations between STX3 protein expression levels and clinicopathological variables demonstrated that high STX3 protein expression in tumor tissue had a significant correlation with tumor location, but it did not significantly correlate with pathological lymph node involvement or advanced pathological stage (Table S2). OS was significantly shorter in the high STX3 group compared to the low STX3 group (HR: 1.79, 95% CI: 1.01–3.19, *p* = 0.049; Figure 2C). DFS between the two groups was not significantly different (HR: 1.57, 95% CI: 0.95–2.58, *p* = 0.078; Figure S3).

### 3.3 STX3 mRNA expression in ESCC cell lines

Due to there being no difference in *STX3* mRNA expression by tumor differentiation in the *STX3* mRNA dataset, we examined *STX3* mRNA expression levels in 21 ESCC cell lines. The levels of *STX3* mRNA expression were higher in 12 ESCC cell lines than the control non‐cancerous epithelial cell line (Hel‐1A), and no differences in STX3 mRNA expression caused by variations in ESCC cell differentiation in the remaining cell lines (Figure S4).

### 3.4 STX3 knockdown efficacy by siRNAs

We transfected KYSE70 with control (siControl) or STX3‐specific (siSTX3) siRNAs. Effective knockdown of *STX3* was confirmed by qPCR analysis, which demonstrated specific reduction of *STX3* mRNA by approximately 60% in KYSE70 cells (Figure S5A).

### 3.5 Western blot and Simple Western assays

We performed conventional western blot assays and confirmed that STX3 protein expression was suppressed in siSTX3‐expressing KYSE70 cells compared to the levels in untransfected or siControl‐transfected cells (Figure S5B). To evaluate the expression of cancer‐related signaling proteins and their phosphorylated forms in KYSE70 cells, we used the Simple Western system. Representative digital blots (Figure S6A) and quantification of protein expression levels (Figure S6B) in untranfected, siControl and siSTX3‐transfected cells is shown. *STX3* silencing affected elements within the PI3K/Akt/mTOR pathway; p‐PI3K and p‐Akt expression were decreased by siSTX3 expression, however, p‐mTOR expression was unaffected. In the ERK1/2 pathway, expression of p‐c‐RAF and p‐MEK1/2 was decreased by siSTX3 expression, but p‐ERK1/2 was unaffected. Finally, in the Wnt/β catenin pathway, expression of Wnt5a/b, p‐LRP6 and β catenin were unaffected.

## 4 DISCUSSION

In the present study, we investigated the prognostic capabilities of both STX3 mRNA and protein levels in ESCC across two independent patient cohorts. We demonstrated that the patients with ESCC who had elevated *STX3* mRNA and protein expression levels experienced significantly shorter OS, suggesting that STX3, either at the genetic or protein level, could serve as a prognostic marker in ESCC following esophagectomy. This study is, to our knowledge, the first to assess the prognostic value of STX3 in patients with ESCC.

The syntaxin family consists of 16 members. However, the physiological and pathological roles of all members of the syntaxin family remain to be elucidated.^26^ In breast cancer, STX3 has been identified as a promoter of growth in human breast cancer cells, and its presence in tumor tissues could act as an indicator of a poor prognosis.^14^ In ESCC, a previous report showed that STX6 was upregulated and significantly associated with tumor size, histologic differentiation, lymph node involvement, and the extent of tumor invasion; in addition, STX6 activity was associated with ESCC progression.^27^ However, until now, there have been no reports on the expression and clinical relevance of STX3 in ESCC.

The aim of this study was to examine the association between *STX3* mRNA expression in surgically resected ESCC tissues and clinicopathologic factors. The results showed that high STX3 mRNA expression was correlated with the presence of lymph node metastasis and advanced pathologic stage. This suggests that STX3 contributes to the malignant progression of ESCC. Conversely, primary tumor factors such as size, depth or location were not significantly associated with high STX3 mRNA expression. For these patients, postoperative treatment, follow‐up planning, and prognostic prediction were generally implemented according to the TNM classification system. However, this system does not reflect biological characteristics of individual cancers and sometimes fails to accurately predict the postoperative outcome.^28^, ^29^ High *STX3* mRNA expression in surgical specimens could better identify high‐risk patients who could be eligible for more aggressive treatments.^30^

In our subgroup analysis, *STX3* mRNA levels were a prognostic factor for OS in patients ≥65 years old, those who did not receive NAC, and those with differentiated tumors. However, the results of our subgroup analysis should be interpreted with discretion given the small number of cases and the altered proportions of the two groups.

Similarly, we demonstrated that high STX3 protein expression via IHC in TMAs was significantly associated with shorter OS. The finding that STX3 expression at the protein level as well as the mRNA level could be a prognostic marker has some advantages. In general, determining protein expression by IHC is easier than measuring mRNA expression.^31^ In this study, tissue specimens used in the TMAs were samples from ESCC patients who had not undergone any treatments before surgery. Therefore, STX3 protein expression was detected by IHC using surgically resected specimens without shrinkage or disappearance of tumor. Additionally, STX3 protein expression in pretreatment biopsy tissues obtained by endoscopic surveillance could also be useful for predicting prognosis.

We found no association between tumor differentiation and *STX3* mRNA expression in surgical specimens. However, surgical specimens have some problems, such as the effect of neoadjuvant chemotherapy and heterogeneity.^32^ Therefore, we investigated the *STX3* mRNA expression levels across ESCC cell lines that exhibit a range of differentiation degrees. We found that *STX3* mRNA expression was increased in both differentiated and undifferentiated ESCC cell lines. This suggests that *STX3* mRNA expression could be a prognostic factor for ESCC with any level of differentiation.

Nan et al. demonstrated that STX3 activates the Akt/mTOR signaling pathway in breast cancer by interacting with PTEN, leading to its ubiquitination and degradation, which in turn stimulates the PI3K/Akt signaling pathway.^14^ However, we found that phosphorylation of mTOR was not suppressed by STX3 knockdown in ESCC cells whereas phosphorylation of PI3K and Akt was slightly decreased after STX3 knockdown. Neither of Phosphorylation of ERK1/2 nor expression of Wnt/β‐catenin were suppressed after STX3 knockdown. These findings suggested that STX3 had only a limited impact on intracellular signaling pathways in ESCC.

Our findings on STX3 could translate into clinical applications for ESCC. For instance, patients with high STX3 mRNA or protein expression in biopsy or surgical resected specimens would benefit from intensive perioperative chemotherapy and postoperative monitoring. If the malignant functions of STX3 in ESCC can be elucidated, it might become a therapeutic target through the development of nucleic acid and/or antibody drugs.^33^, ^34^

There are a few limitations to this study. First, this study was of a retrospective nature and involved a small sample size. Therefore, we need to conduct a prospective study in a larger patient cohort to assure the validity of STX3 as a prognostic marker. Second, since we considered that STX3 plays an important role in a prognostic biomarker of ESCC, rather than oncogenic functions, functional assays to explore the oncogenic potential of STX3 in ESCC cells were not conducted in this study. We need to perform many further experiments to elucidate the molecular mechanisms of STX3, which could lead to an explanation of the correlation between high STX3 expression and poor prognosis.

In conclusion, we found a potential clinical utility for both STX3 mRNA and protein levels as a poor prognostic marker in ESCC. We would consider the clinical application of this molecular biomarker to be a means of improving the treatment of ESCC.

## AUTHOR CONTRIBUTIONS

**Takahiro Shinozuka:** Conceptualization (lead); data curation (lead); formal analysis (lead); methodology (lead); resources (lead); software (lead); visualization (lead); writing – original draft (lead). **Mitsuro Kanda:** Conceptualization (supporting); writing – original draft (supporting). **Yusuke Sato:** Supervision (supporting). **Dai Shimizu:** Supervision (supporting). **Chie Tanaka:** Supervision (supporting). **Shinichi Umeda:** Supervision (supporting). **Yoshikuni Inokawa:** Supervision (supporting). **Norifumi Hattori:** Supervision (supporting). **Masamichi Hayashi:** Supervision (supporting). **Goro Nakayama:** Supervision (supporting). **Yasuhiro Kodera:** Supervision (lead).

## CONFLICT OF INTEREST STATEMENT

All authors declare that they have no commercial conflicts of interest to disclose.

## Supporting information


Figure S1.

Figure S2.

Figure S3.

Figure S4.

Figure S5.

Figure S6.

Table S1.

Table S2.

## Data Availability

The data underlying this article will be shared on a reasonable request to the corresponding author.

## References

[1] Siegel RL , Miller KD , Wagle NS , Jemal A . Cancer statistics, 2023. CA Cancer J Clin. 2023;73:17‐48. 36633525 10.3322/caac.21763

[2] Yusupbekov A , Kanda M , Usmanov B , et al. Surveillance of esophageal cancer in the Republic of Uzbekistan from 2000 to 2018. Asian Pac J Cancer Prev. 2020;21:2281‐2285. 32856856 10.31557/APJCP.2020.21.8.2281 PMC7771931

[3] Shinozuka T , Kanda M , Shimizu D , et al. Prognostic value of a modified albumin‐bilirubin score designed for patients with esophageal squamous cell carcinoma after radical resection. Ann Surg Oncol. 2022;29:4889‐4896. 35381933 10.1245/s10434-022-11654-6

[4] Baba H , Kanda M , Sawaki K , et al. SLC7A9 as a potential biomarker for lymph node metastasis of esophageal squamous cell carcinoma. Ann Surg Oncol. 2022;29:2699‐2709. 34773193 10.1245/s10434-021-11001-1

[5] Nakamura S , Kanda M , Koike M , et al. KCNJ15 expression and malignant behavior of esophageal squamous cell carcinoma. Ann Surg Oncol. 2020;27:2559‐2568. 32052303 10.1245/s10434-019-08189-8

[6] Baba H , Kanda M , Sawaki K , et al. PRAME expression as a potential biomarker for hematogenous recurrence of esophageal squamous cell carcinoma. Anticancer Res. 2019;39:5943‐5951. 31704819 10.21873/anticanres.13799

[7] Janecke AR , Liu X , Adam R , et al. Pathogenic STX3 variants affecting the retinal and intestinal transcripts cause an early‐onset severe retinal dystrophy in microvillus inclusion disease subjects. Hum Genet. 2021;140:1143‐1156. 33974130 10.1007/s00439-021-02284-1 PMC8263458

[8] Pattu V , Qu B , Schwarz EC , et al. SNARE protein expression and localization in human cytotoxic T lymphocytes. Eur J Immunol. 2012;42:470‐475. 22120889 10.1002/eji.201141915

[9] Giovannone AJ , Winterstein C , Bhattaram P , et al. Soluble syntaxin 3 functions as a transcriptional regulator. J Biol Chem. 2018;293:5478‐5491. 29475951 10.1074/jbc.RA117.000874 PMC5900775

[10] Sanchez E , Gonzalez EA , Moreno DS , et al. Syntaxin 3, but not syntaxin 4, is required for mast cell‐regulated exocytosis, where it plays a primary role mediating compound exocytosis. J Biol Chem. 2019;294:3012‐3023. 30563839 10.1074/jbc.RA118.005532 PMC6398129

[11] Kinghorn K , Gill A , Marvin A , et al. A defined clathrin‐mediated trafficking pathway regulates sFLT1/VEGFR1 secretion from endothelial cells. Angiogenesis. 2023. doi:10.1101/2023.01.27.525517 PMC10881643 37695358

[12] Day P , Riggs KA , Hasan N , et al. Syntaxins 3 and 4 mediate vesicular trafficking of α5β1 and α3β1 integrins and cancer cell migration. Int J Oncol. 2011;39:863‐871. 21720706 10.3892/ijo.2011.1101

[13] Motoike S , Taguchi K , Harada K , et al. Syntaxin 3 interacts with serotonin transporter and regulates its function. J Pharmacol Sci. 2021;145:297‐307. 33712280 10.1016/j.jphs.2021.01.007

[14] Nan H , Han L , Ma J , Yang C , Su R , He J . STX3 represses the stability of the tumor suppressor PTEN to activate the PI3K‐Akt‐mTOR signaling and promotes the growth of breast cancer cells. Biochim Biophys Acta Mol Basis Dis. 2018;1864:1684‐1692. 29408595 10.1016/j.bbadis.2018.01.031

[15] Chen Y , Shen L , Chen B , et al. The predictive prognostic values of CBFA2T3, STX3, DENR, EGLN1, FUT4, and PCDH7 in lung cancer. Ann Transl Med. 2021;9:843. 34164477 10.21037/atm-21-1392 PMC8184469

[16] Escrig Sos J , Gómez Quiles L , Maiocchi K . The 8th edition of the AJCC‐TNM classification: New contributions to the staging of esophagogastric junction cancer. Cir Esp (Engl Ed). 2019;97:432‐437. 31029372 10.1016/j.ciresp.2019.03.006

[17] Kanda M , Koike M , Iwata N , et al. An open‐label single‐arm phase II study of treatment with neoadjuvant S‐1 plus cisplatin for clinical stage III squamous cell carcinoma of the esophagus. Oncologist. 2020;25:e1650‐e1654. 32557987 10.1634/theoncologist.2020-0546 PMC7648369

[18] Shimizu D , Koike M , Kanda M , et al. Newly developed primary malignancies in long‐term survivors who underwent curative esophagectomy for squamous cell carcinoma of the esophagus. Surg Today. 2021;51:153‐158. 32638131 10.1007/s00595-020-02072-w

[19] Umeda S , Kanda M , Shimizu D , et al. Lysosomal‐associated membrane protein family member 5 promotes the metastatic potential of gastric cancer cells. Gastric Cancer. 2022;25:558‐572. 35226222 10.1007/s10120-022-01284-y

[20] Baba H , Kanda M , Sawaki K , et al. PRAME as a potential biomarker for liver metastasis of gastric cancer. Ann Surg Oncol. 2020;27:2071‐2080. 31659640 10.1245/s10434-019-07985-6

[21] Sasahara M , Kanda M , Shimizu D , et al. Tissue RNFT2 expression levels are associated with peritoneal recurrence and poor prognosis in gastric cancer. Anticancer Res. 2021;41:609‐617. 33517265 10.21873/anticanres.14812

[22] Sato Y , Motoyama S , Wakita A , et al. TLR3 expression status predicts prognosis in patients with advanced thoracic esophageal squamous cell carcinoma after esophagectomy. Am J Surg. 2018;216:319‐325. 29395019 10.1016/j.amjsurg.2018.01.038

[23] Baba H , Kanda M , Sato Y , et al. Expression and malignant potential of B4GALNT4 in esophageal squamous cell carcinoma. Ann Surg Oncol. 2020;27:3247‐3256. 32253672 10.1245/s10434-020-08431-8

[24] Ueda S , Kanda M , Sato Y , et al. Chromobox 2 expression predicts prognosis after curative resection of oesophageal squamous cell carcinoma. Cancer Genomics Proteomics. 2020;17:391‐400. 32576584 10.21873/cgp.20198 PMC7367605

[25] Shinozuka T , Kanda M , Ito S , et al. D2 lymph node dissection confers little benefit on the overall survival of older patients with resectable gastric cancer: a propensity score‐matching analysis of a multi‐institutional dataset. Surg Today. 2020;50:1434‐1442. 32451713 10.1007/s00595-020-02021-7

[26] Zhao ZZ , Duffy DL , Thomas SA , Martin NG , Hayward NK , Montgomery GW . Polymorphisms in the syntaxin 17 gene are not associated with human cutaneous malignant melanoma. Melanoma Res. 2009;19:80‐86. 19209086 10.1097/CMR.0b013e328322fc45 PMC3665505

[27] Du J , Liu X , Wu Y , et al. Essential role of STX6 in esophageal squamous cell carcinoma growth and migration. Biochem Biophys Res Commun. 2016;472:60‐67. 26906622 10.1016/j.bbrc.2016.02.061

[28] Wang C , Wang J , Chen Z , Gao Y , He J . Immunohistochemical prognostic markers of esophageal squamous cell carcinoma: a systematic review. Chin J Cancer. 2017;36:65. 28818096 10.1186/s40880-017-0232-5 PMC5561640

[29] Xiao Z , Jia Y , Jiang W , Wang Z , Zhang Z , Gao Y . FOXM1: a potential indicator to predict lymphatic metastatic recurrence in stage IIA esophageal squamous cell carcinoma. Thorac Cancer. 2018;9:997‐1004. 29877046 10.1111/1759-7714.12776 PMC6068428

[30] Kanda M , Koike M , Shimizu D , et al. Characteristics associated with nodal and distant recurrence after radical esophagectomy for squamous cell carcinoma of the thoracic esophagus. Ann Surg Oncol. 2020;27:3195‐3205. 32246314 10.1245/s10434-020-08433-6

[31] Miyoshi J , Zhu Z , Luo A , et al. A microRNA‐based liquid biopsy signature for the early detection of esophageal squamous cell carcinoma: a retrospective, prospective and multicenter study. Mol Cancer. 2022;21:44. 35148754 10.1186/s12943-022-01507-x PMC8832722

[32] Uno Y , Kanda M , Sato Y , et al. Expression, function, and prognostic value of MAGE‐D4 protein in esophageal squamous cell carcinoma. Anticancer Res. 2019;39:6015‐6023. 31704827 10.21873/anticanres.13807

[33] Kanda M , Shimizu D , Sawaki K , et al. Therapeutic monoclonal antibody targeting of neuronal pentraxin receptor to control metastasis in gastric cancer. Mol Cancer. 2020;19:131. 32847597 10.1186/s12943-020-01251-0 PMC7448342

[34] Kanda M , Kasahara Y , Shimizu D , et al. Amido‐bridged nucleic acid‐modified antisense oligonucleotides targeting SYT13 to treat peritoneal metastasis of gastric cancer. Mol Ther Nucleic Acids. 2020;22:791‐802. 33230476 10.1016/j.omtn.2020.10.001 PMC7644579

