# Supplementary figures and images for: Increased STX3 transcript and protein levels were associated with poor prognosis in two independent cohorts of esophageal squamous cell carcinoma patients
Source: Cancer Med. 2023 Nov 28;12(24):22185–95. doi: 10.1002/cam4.6770 (PMC10757105; doi:10.1002/cam4.6770)

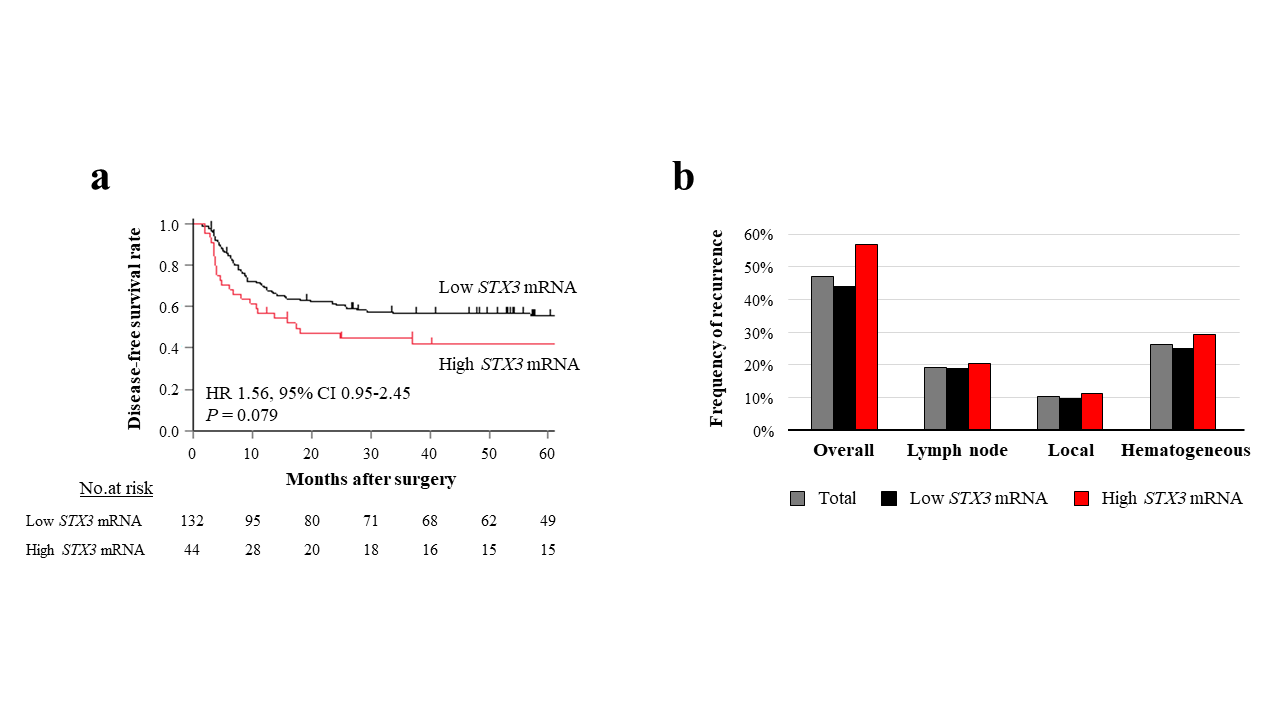

Supplement: Supplementary file 1 — Figure S1. [file CAM4-12-22185-s007.tif]

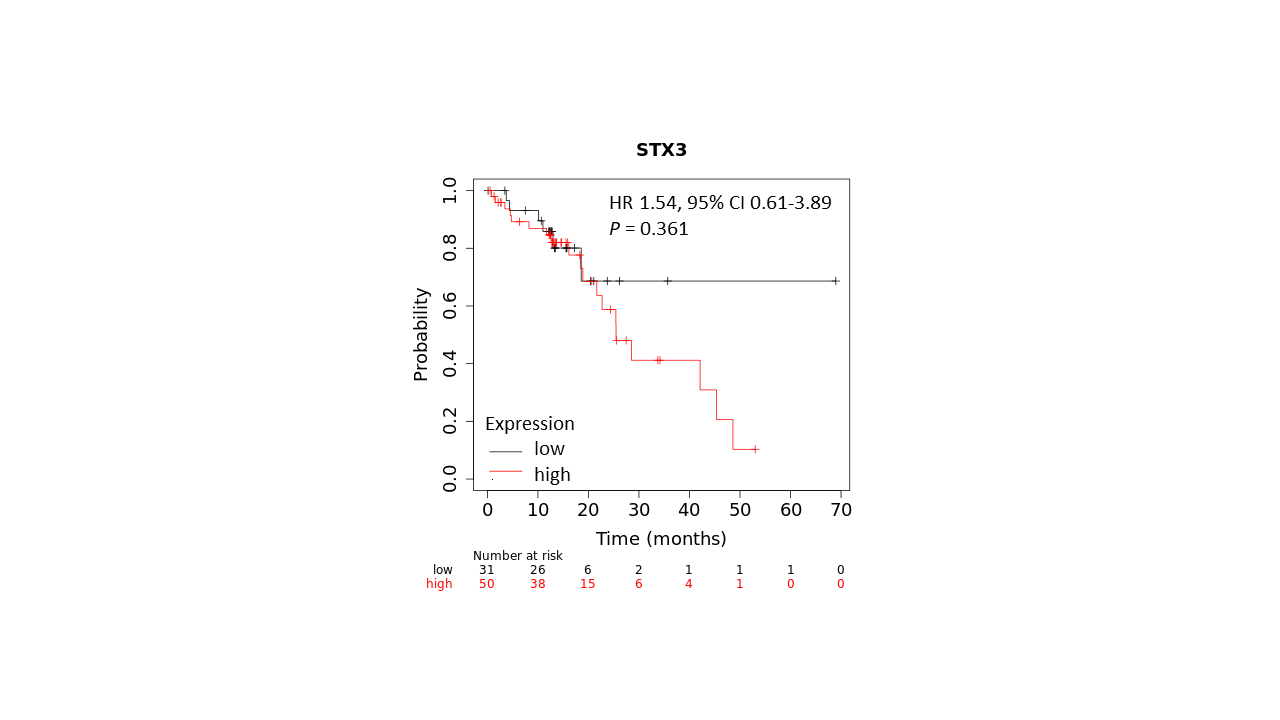

Supplement: Supplementary file 2 — Figure S2. [file CAM4-12-22185-s002.tif]

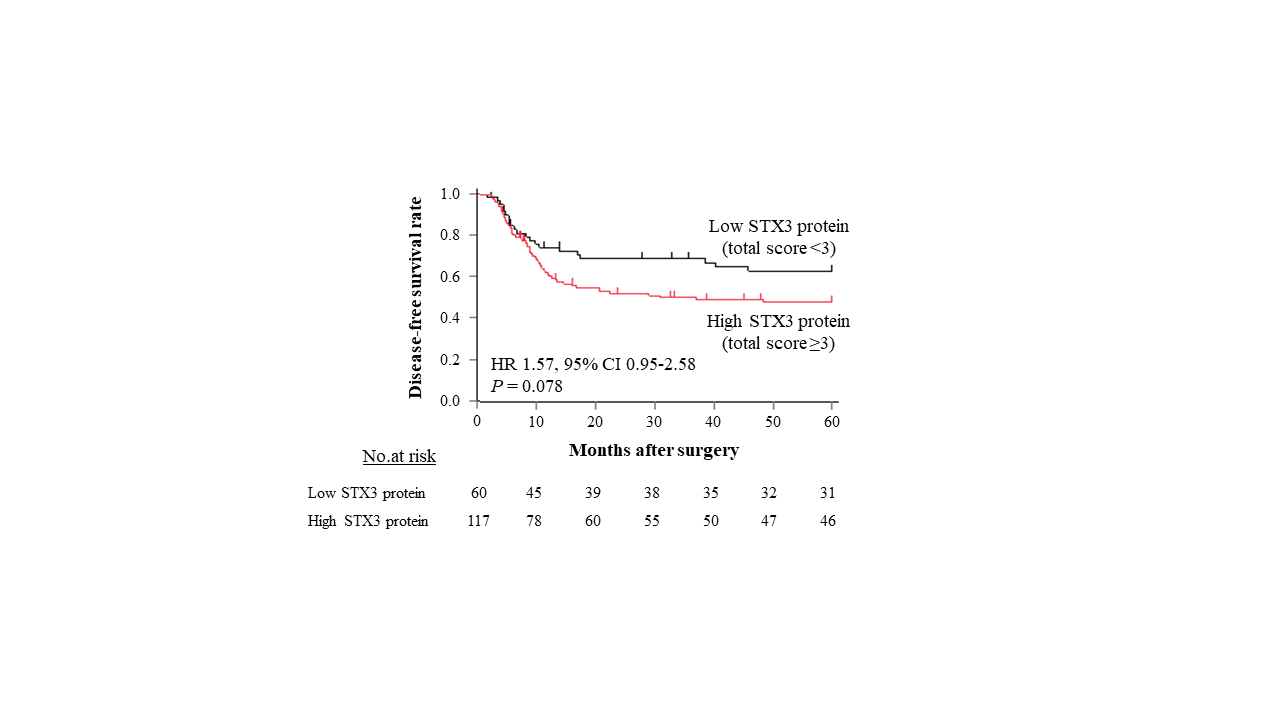

Supplement: Supplementary file 3 — Figure S3. [file CAM4-12-22185-s008.tif]

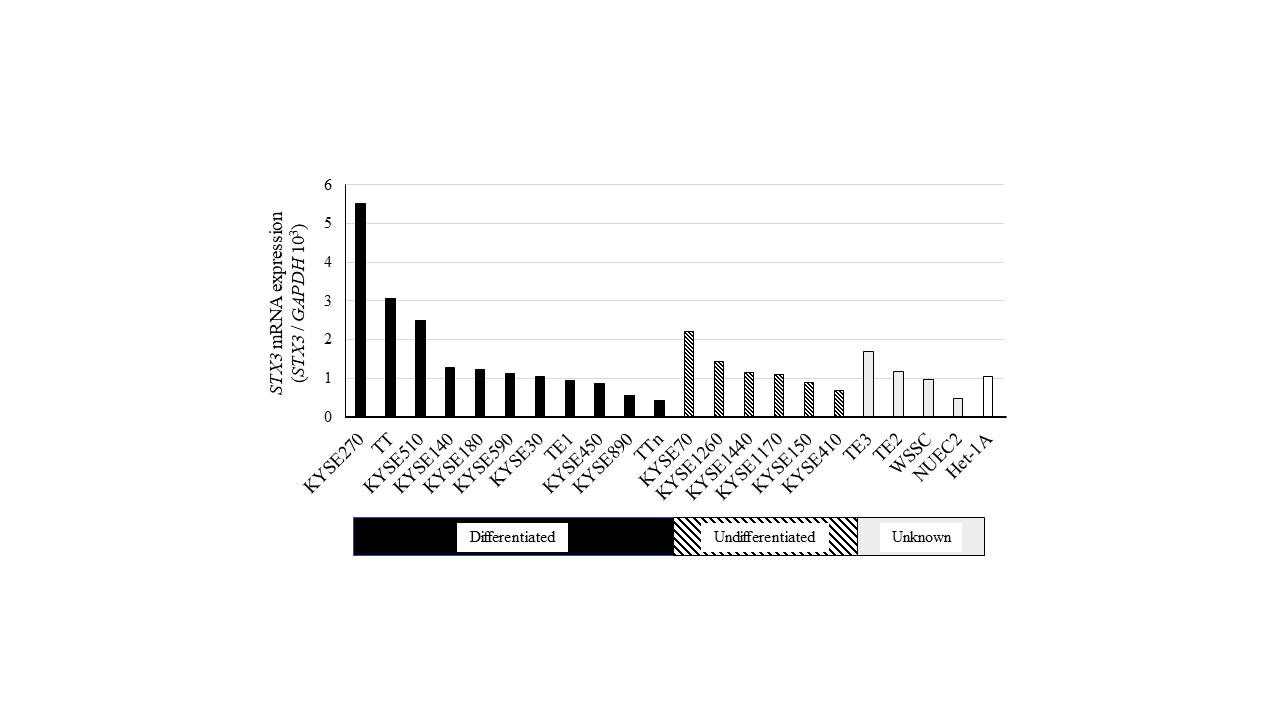

Supplement: Supplementary file 4 — Figure S4. [file CAM4-12-22185-s004.tif]

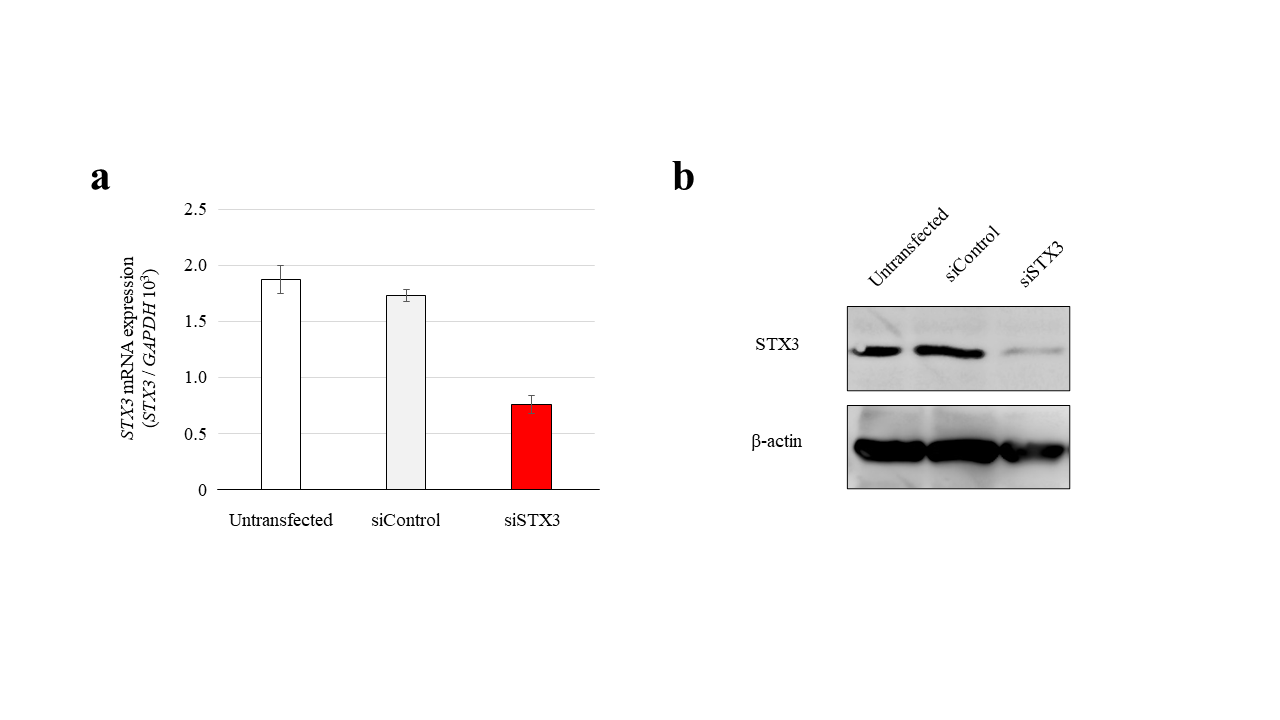

Supplement: Supplementary file 5 — Figure S5. [file CAM4-12-22185-s001.tif]

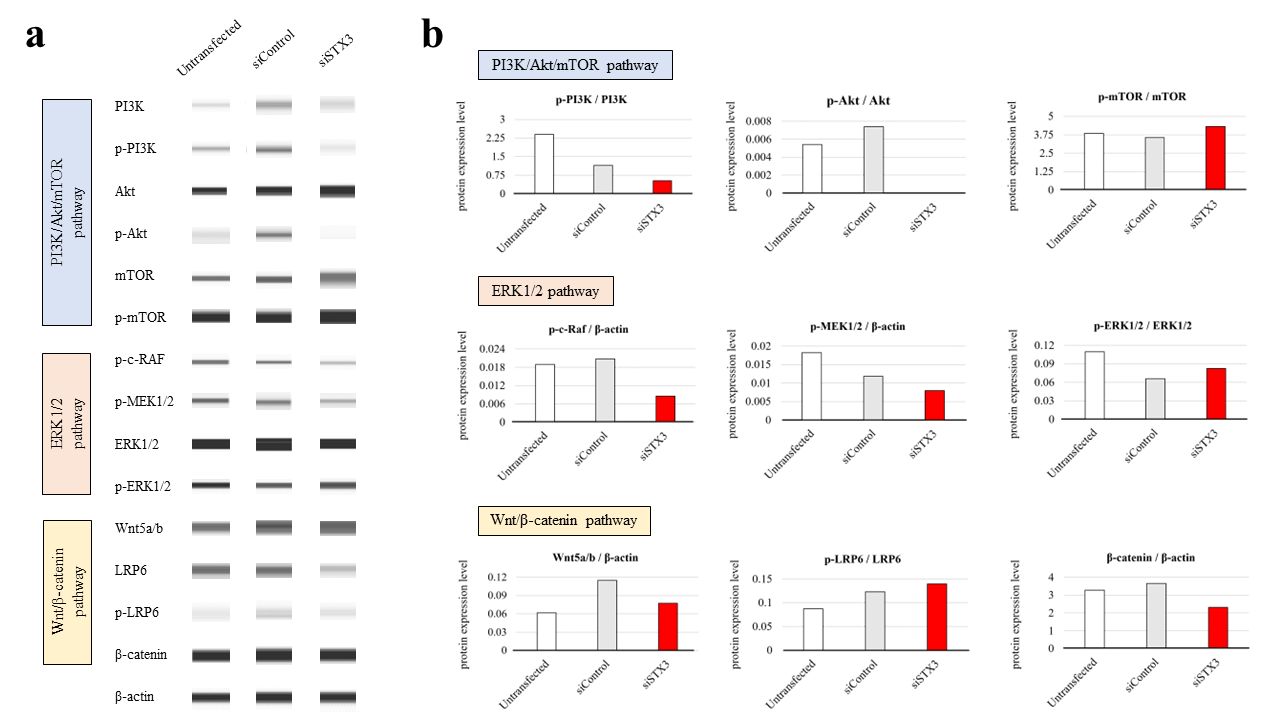

Supplement: Supplementary file 6 — Figure S6. [file CAM4-12-22185-s006.tif]
