# Supplementary material for: Increased STX3 transcript and protein levels were associated with poor prognosis in two independent cohorts of esophageal squamous cell carcinoma patients
Source: Cancer Med. 2023 Nov 28;12(24):22185–95. doi: 10.1002/cam4.6770 (PMC10757105; doi:10.1002/cam4.6770)
Supplement: Supplementary file 7 — Table S1. [file CAM4-12-22185-s005.docx]

| **Gene** | **Experiment** | **Type** | **Sequence (5′ - 3′)** | **Product size** | **Annealing temperature** |
| --- | --- | --- | --- | --- | --- |
| ***STX3*** | qRT-PCR | forward | ACACGGCTTTTATGGACGAG | 189 bp | 60 °C |
|  |  | reverse | CGTTGTTGGCCCTTTTCTTA |  |  |
|  | siRNA | si*STX3*-1 | GGAAGAAAUUGAUAAUUAUTT |  |  |
|  |  | si*STX3*-2 | GGCAUUUUAGCAUUGAUUATT |  |  |
|  |  | si*STX3*-3 | CGAAAAAAGCUGUGAAAUACC |  |  |
| **Control** | siRNA | siControl | GCAAACAUCCCAGAGGUAU |  |  |
| ***GAPDH*** | qRT-PCR | forward | GAAGGTGAAGGTCGGAGTC | 226 bp | 60 °C |
|  |  | probe | CAAGCTTCCCGTTCTCAGCC |  |  |
|  |  | reverse | GAAGATGGTGATGGGATTTC |  |  |

**Supplementary Table 1.** Sequences of primers and siRNAs.

*STX3* Syntaxin 3, *GAPDH* glyceraldehyde-3-phosphate dehydrogenase, *qRT-PCR* quantitative reverse-transcription polymerase chain reaction, *siRNA* small interfering RNA, *bp* base pair.
