# Supplementary material for: Increased STX3 transcript and protein levels were associated with poor prognosis in two independent cohorts of esophageal squamous cell carcinoma patients
Source: Cancer Med. 2023 Nov 28;12(24):22185–95. doi: 10.1002/cam4.6770 (PMC10757105; doi:10.1002/cam4.6770)
Supplement: Supplementary file 8 — Table S2. [file CAM4-12-22185-s003.docx]

**Supplementary Table 2.** Association between the expression of STX3 protein and clinicopathological parameters of 177 patients with esophageal squamous cell carcinoma.

| Parameters | Low STX3 protein (*n*=60) | High STX3 protein (*n*=117) | *P* |
| --- | --- | --- | --- |
| Age  < 65 years  ≥ 65 years | 22  38 | 51  66 | 0.422 |
| Sex  Male  Female | 52  8 | 101  16 | 1.000 |
| Brinkman index  < 500  ≥ 500 | 23  37 | 35  82 | 0.311 |
| Tumor location  Ut / Mt  Lt / Ae | 35  25 | 87  30 | 0.039 |
| Pathological T factor  T1 / T2  T3 / T4 | 12  48 | 19  98 | 0.537 |
| Lymph node metastasis  Absent  Present | 16  33 | 44  84 | 0.861 |
| Pathological stage  I / II  III / IV | 18  42 | 36  81 | 1.000 |
| Tumor differentiation  Differentiated  Undifferentiated | 40  20 | 80  37 | 0.866 |

STX3, Syntaxin 3.
